# Thyroid autoimmunity is associated with fertilization impairment and follicular fluid exosomal miRNA alterations in euthyroid women undergoing IVF-ET

**DOI:** 10.3389/fimmu.2026.1870650

**Published:** 2026-07-10

**Authors:** Lanping Zhong, Yuting Luo, Jiani Zhu, Xin Li, Yuhong Niu, Ji-Gan Wang, Xiaomin Kang

**Affiliations:** 1 Department of Reproductive Medicine, The First People’s Hospital of Yunnan Province, Kunming, Yunnan, China; 2 The First People’s Hospital of Yunnan Province, The Affiliated Hospital of Kunming University of Science and Technology, Kunming, China; 3 NHC Key Laboratory of Preconception Health Birth in Western China, Kunming, China; 4 Department of Clinical Nutrition, The First People’s Hospital of Yunnan Province, The Affiliated Hospital of Kunming University of Science and Technology, Kunming, Yunnan, China; 5 Department of Pediatrics, Maternal and Child Health Hospital of Guangxi Zhuang Autonomous Region, Nanning, China; 6 The Kunming Medical University, Kunming, Yunnan, China; 7 Yunnan Provincial Key Laboratory of Public Health and Biosafety, Kunming, China

**Keywords:** euthyroid, *in vitro* fertilization–embryo transfer, miRNA, ollicular fluid exosomes, thyroid autoimmunity

## Abstract

**Objective:**

To investigate the impact of thyroid autoimmunity (TAI) on fertilization outcomes in euthyroid infertile women undergoing *in vitro* fertilization–embryo transfer (IVF-ET), and to identify key follicular fluid exosomal miRNAs associated with TAI.

**Methods:**

A total of 4,332 infertile women aged 20–40 years undergoing IVF-ET at the First People’s Hospital of Yunnan Province between January and October 2024 were retrospectively enrolled. TAI was defined as TPOAb >34 IU/mL and/or TgAb >115 IU/mL. Fertilization rates were compared using the Wilcoxon rank-sum test, and the association between TAI and fertilization outcomes was assessed using generalized linear models. For mechanistic analysis, follicular fluid samples from 10 euthyroid TAI-positive and 10 euthyroid TAI-negative women were subjected to exosomal miRNA sequencing. TAI-related miRNAs were identified by integrating WGCNA and differential expression analysis (edgeR), followed by GO and KEGG enrichment analyses. Key miRNAs were screened using LASSO and SVM, and their diagnostic performance was evaluated by ROC analysis. Target genes were predicted using miRWalk and used to construct a miRNA–mRNA regulatory network with subsequent functional enrichment analysis.

**Results:**

Among the 4,332 infertile women undergoing IVF-ET, 565 were TAI-positive and 3,767 were TAI-negative. The fertilization rate was significantly lower in the TAI-positive group, and TAI was identified as an independent risk factor for impaired fertilization in euthyroid women. Exosomal miRNA sequencing of follicular fluid identified 70 differentially expressed miRNAs, and 17 candidate miRNAs were obtained by integrating WGCNA results. Further screening using LASSO and SVM identified four key miRNAs: hsa-miR-6799-3p, hsa-miR-6836-3p, hsa-miR-4449, and hsa-miR-3162-5p, all of which demonstrated good diagnostic performance (AUC > 0.7). Functional enrichment analysis of target genes suggested that these miRNAs may be involved in TAI-related reproductive impairment through pathways such as cell–matrix adhesion and mTOR signaling.

**Conclusion:**

TAI is independently associated with impaired fertilization outcomes in euthyroid infertile women undergoing IVF-ET and is accompanied by altered expression profiles of follicular fluid exosomal miRNAs. hsa-miR-6799-3p, hsa-miR-6836-3p, hsa-miR-4449, and hsa-miR-3162-5p may serve as potential molecular biomarkers for TAI-related reproductive dysfunction, providing insights into underlying mechanisms and potential diagnostic targets.

## 1 Introduction

Infertility is a common reproductive health issue among women of reproductive age, significantly impairing quality of life and imposing substantial social and familial burdens (1, 2). In recent years, in addition to classical factors such as age, ovarian reserve, and embryo quality, increasing attention has been paid to the roles of immune and endocrine factors in the development of infertility and outcomes of assisted reproductive technologies (ART) (3).

Thyroid autoimmunity (TAI), characterized by the presence of thyroid peroxidase antibodies (TPOAb) and/or thyroglobulin antibodies (TgAb), represents one of the most common thyroid immune abnormalities in women of reproductive age. Previous studies have suggested that TAI may be associated with impaired female reproductive function, adverse pregnancy outcomes, and poor ART outcomes. However, whether TAI independently affects ART/IVF outcomes in euthyroid women remains controversial. On the one hand, systematic reviews and meta-analyses indicate that TAI may have detrimental effects on certain ART outcomes; on the other hand, some studies focusing specifically on euthyroid women report no clear impact of TAI on IVF/ICSI pregnancy outcomes, highlighting the need for further investigation (4, 5).

Regarding the underlying mechanisms, the “ovarian follicle hypothesis” has been proposed (6), suggesting that thyroid autoantibodies may cross the blood–follicle barrier and enter the follicular fluid microenvironment, thereby affecting oocyte maturation, fertilization, and embryo development. Busnelli A (7) reported that thyroid autoantibodies could be detected in the follicular fluid of euthyroid infertile women with TAI and were associated with poorer fertilization, embryo quality, and pregnancy outcomes. Subsequently, Medenica et al. demonstrated a significant correlation between thyroid autoantibody levels in serum and follicular fluid, further supporting their potential role in influencing ART outcomes (8).

Follicular fluid represents a critical local microenvironment for oocyte development and maturation, and its composition directly reflects the metabolic, paracrine, and immune status within the follicle. In recent years, exosomes and their cargo, particularly microRNAs (miRNAs), have emerged as key mediators of intercellular communication and a research hotspot in reproductive medicine. Accumulating evidence indicates that extracellular/exosomal miRNAs in human follicular fluid are closely associated with fertilization status, day 3 embryo quality, and oocyte competence, suggesting their potential as molecular biomarkers for assessing follicular microenvironment and reproductive outcomes (9, 10). In addition, review studies have highlighted the involvement of miRNAs in oocyte maturation and female reproductive regulation, underscoring their mechanistic and biomarker potential (11).

Therefore, this study aimed to first evaluate the association between TAI and fertilization outcomes in euthyroid infertile women undergoing IVF-ET. Furthermore, based on follicular fluid exosomal miRNA sequencing, we integrated weighted gene co-expression network analysis (WGCNA), differential expression analysis, and machine learning approaches to identify key TAI-related miRNAs and explore their potential biological pathways, thereby providing insights into the mechanisms of TAI-related reproductive impairment and identifying potential molecular biomarkers.

## 2 Materials and methods

### 2.1 Impact of TAI on fertilization in infertile women

The data of infertility patients who underwent IVF-ET in The First People’s Hospital of Yunnan Province from January 2024 to October 2024. A total of 4332 infertile women were considered potential candidates for inclusion in this study. TPOAb>34IU/ml, or TgAb>115IU/ml were considered to indicate the presence of TAI. A Wilcoxon test was conducted to analyze intergroup differences. Key risk factors influencing fertilization rate were identified through simple and multiple linear regression to assess combined effect on fertilization rate.

#### 2.1.1 Inclusion criteria

1. Age 23–40 years;
2. First IVF/ICSI cycle due to tubal factor infertility, unexplained infertility, or male factor infertility;
3. Euthyroid status (TSH 0.35–4.0 mIU/L, normal FT3 and FT4, and no history of thyroid hormone therapy);
4. Positive thyroid peroxidase antibody (TPOAb) and/or thyroglobulin antibody (TgAb);
5. Normal ovarian reserve (FSH <10 IU/L, AMH ≥1.1 ng/mL, antral follicle count ≥5);
6. Regular menstrual cycles;
7. Body mass index (BMI) <28 kg/m²;
8. Normal uterine cavity morphology.

#### 2.1.2 Exclusion criteria

1. Polycystic ovary syndrome (PCOS), endometriosis, diminished ovarian reserve (DOR), or premature ovarian insufficiency (POI);
2. Azoospermia or chromosomal abnormalities in the male partner;
3. History of ≥1 previous IVF/ICSI cycle;
4. Recurrent miscarriage (≥2 consecutive losses);
5. History of thyroid disease or other autoimmune diseases (e.g., systemic lupus erythematosus, antiphospholipid syndrome);
6. Diabetes mellitus, pituitary or adrenal disorders;
7. Uterine malformations or intrauterine adhesions;
8. Chromosomal abnormalities in either partner;
9. Severe hepatic or renal dysfunction, or cardiovascular disease;
10. Use of oral contraceptives or pregnancy within the past 3 months.

### 2.2 Sample collection and RNA extraction and sequencing

We selected FF samples from 10 euthyroid women with TAI and 10 euthyroid women without TAI, all of whom were among 4332 infertile women undergoing IVF-ET at The First People’s Hospital of Yunnan Province. FF samples were obtained from each participant for exosomal miRNA sequencing, with ethical approval and informed consent secured. Moreover, RNA from total samples was isolated and purified using TRIzol (Invitrogen, CA, USA). The amount and purity of total RNA was quality controlled using NanoDrop ND-1000 (NanoDrop, Wilmington, DE, USA). RNA integrity was examined by Bioanalyzer 2100 (Agilent, CA, USA) and verified by agarose electrophoresis setting concentration > 50ng/μL, RIN value > 7.0, OD260/280 > 1.8, and total RNA > 1μg. The mRNA with polyadenylate (PolyA) was specifically captured by two rounds of purification using dynabeads oligo (dT) 25-61005 magnetic beads (Thermo Fisher, USA). The captured mRNA was fragmented at 94 °C for 5-7 minutes using magnesium RNA fragmentation module (NEB, cat.e6150, USA), and then the fragmented RNA was passed through reverse transcriptase (Invitrogen, cat. 1896649, USA) to synthesize cDNA. Duplex synthesis was performed using E. coli DNA polymerase I (NEB, Item m0209, USA) and RNase H (NEB, Item m0297, USA) to convert these DNA and RNA complex duplexes into DNA duplexes. The dUTP solution (Thermo Fisher, Item No. R0133, CA, USA) was doped into the second strand. The ends of the double-stranded DNA were made up to flat ends, an A base was added to each end to enable it to ligate with a connector with T base at the end, and fragment size was screened and purified using magnetic beads. The second strand was digested with UDG enzyme (NEB, item no. m0280, MA, US), and the experimental flow was designed by PCR with pre-denaturation at 95 °C and held for 3 minutes, denaturation at 98 °C for 15s for 8 cycles, annealed to 60 °C for 15s, extension at 72 °C for 30s, and extension at 72 °C for 5 minutes to form a library with fragment size of 300 bp ± 50 bp. Finally, we performed double-end sequencing using illumina Novaseq™ 6000 (LC Bio Technology CO., Ltd. Hangzhou, China) in PE150 sequencing mode according to the standard operation. The exosomal miRNA profiles from the follicular fluid of 10 euthyroid women without TAI and 10 euthyroid women with TAI have been uploaded into Sequence Read Archive (SRA) in NCBI (SRA accession numbers: SRR32920729-SRR32920748).

### 2.3 Screening of hub miRNA

To identify phenotypically related hub miRNA in the miRNA expression matrix, we clustered 20 samples to exclude outliers and performed soft threshold determination on the data. By constructing the co-expression matrix, the neighbor-joining and similarity coefficients between genes were calculated, and a systematic clustering tree between genes was drawn. Then the minimum number of genes per gene module was set according to the criteria of dynamic tree cutting, and MEDissThres was set to 0.2 to merge the similar modules analyzed by dynamic tree cutting. Moreover, we analyzed the correlation between clinical features and modular gene expression in TAI-positive and TAI-negative groups, and identified hub miRNAs associated with TAI positivity based on a significance threshold of P < 0.05 and |r| > 0.4.

### 2.4 Evaluation of differential expression miRNAs between euthyroid women with and without TAI

Differential expression miRNAs were analyzed by using edgeR package (12) for 10 TAI-positive samples and 10 TAI-negative samples in the miRNA expression matrix. Then a threshold was set to screen out differentially expressed miRNAs (DE-miRNAs) at P < 0.05 and |log2fold change (FC)| > 0.5, and the result was visualized by drawing volcano and heat maps. The intersection of hub miRNA and differential expression miRNA was taken using jVenn (http://jvenn.toulouse.inra.fr/app/example.html) to obtain the intersecting miRNA.

### 2.5 Functional enrichment analysis of intersecting miRNA

Gene ontology (GO) statistical and Kyoto encyclopedia of genes and genomes (KEGG) enrichment analyses were performed by using the miEAA database (v2.0, https://ccb-compute2.cs.uni-saarland.de/mieaa2/) for intersecting miRNA with a screening condition of minimum required hits per sub-category was 2 and P < 0.05. The enrichment results were visualized by plotting bar graphs with ggplot2 package (13).

### 2.6 Identification of the critical miRNA associated with TAI in euthyroid women

Firstly, the study based on the expression values of the intersecting miRNA in the miRNA expression matrix for each sample, and combined with the grouping information of the samples to construct LASSO regression to predict the sample classification. The feature miRNA was selected using glmnet package (version4.0-2) (14) with parameters family=“binomial”, type.measure=“class”, nfold = 10 for 10-fold cross-validation. The graphs of gene coefficients and cross-validation error maps were obtained by selecting strongly correlated features. Then, the intersecting genes were ranked by SVM algorithm using e1071 package (version1.7-9) (15). RFE was used to obtain the importance and importance ranking of each gene, as well as to obtain the error rate and accuracy rate of each iteration of the combination. Moreover, the lowest error rate point was selected as the best combination and the corresponding gene was taken out as the feature miRNA. Finally, the critical miRNA was obtained by intersecting the characteristic miRNA screened by LASSO regression analysis and SVM analysis respectively using VennDiagram package (16). The pROC package (17) was used to draw ROC curves to analyze the diagnostic value of critical miRNA to distinguish TAI-positive samples from TAI-negative samples.

### 2.7 Construction of miRNA-mRNA network

The study used miRwalk3.0 database to perform prediction analysis of critical miRNA based on the default parameters binding probability ≥ 0.95, binding site position = 3UTR, and the miRNA-mRNA regulatory pairs were selected. Then, the miRNA-mRNA regulatory network was constructed by using Cytoscape software.

### 2.8 Functional enrichment analysis of mRNA in miRNA-mRNA relational pair

GO and KEGG enrichment analysis of mRNA in the miRNA-mRNA regulatory network was performed by setting the screening condition to p < 0.05 using clusterProfiler package (18). The enrichment results were visualized using ggplot2 package (13).

Written consent was obtained from each patient after a full explanation of the purpose and nature of all procedures used. The study protocol was approved by the Institutional Ethics Committee of the First People’s Hospital of Yunnan Province (**KHLL2023-KY101**).

## 3 Results

### 3.1 TAI as a risk factor for fertilization impairment in euthyroid women undergoing IVF-ET cycles

**Figure 1:**
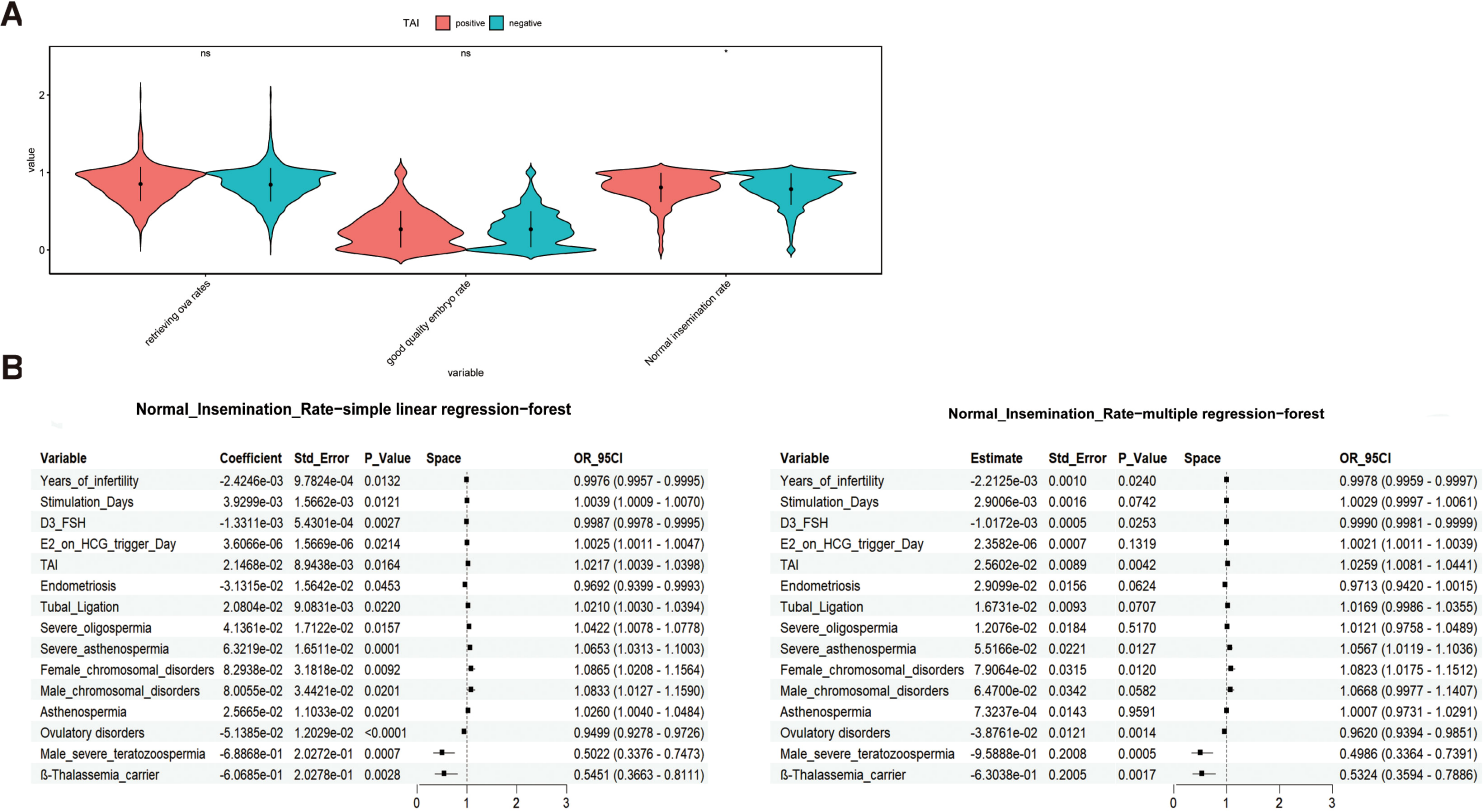
TAI serve as a risk factor for fertilization in euthyroid women undergoing IVF-ET. **(A)** A Wilcoxon test was performed to assess different fertilization in euthyroid women with and without TAI **(B)** TAI as a risk factor for fertilization in euthyroid women undergoing IVF-ET using simple linear regression (left) and multiple regression (right).

As presented in **Table 1**, a total of 4332 infertile women who underwent IVF-ET were enrolled, including 565 with positive TAI and 3767 with negative TAI. The TAI positive group had a mean age of (32.75 ± 5.02) years, BMI of (22.47 ± 2.75), a duration of infertility of (4.39 ± 3.24) years, a day 3 follicle-stimulating hormone (D3_FSH) level of (4.65 ± 3.59) IU/L, an AMH level of (4.17 ± 3.28) ng/mL, and a total AFC of (13.59 ± 5.70). The TAI negative group had a mean age of (32.07 ± 4.85) years, a BMI of (22.24 ± 2.77), a duration of infertility of (4.21 ± 3.12) years, a D3_FSH level of (4.56 ± 7.28) IU/L, an AMH level of (4.60 ± 3.65) ng/mL, and a Total AFC of (14.15 ± 5.62).A Wilcoxon test was used to assess intergroup differences, revealing a significant variation in fertilization rates (**Figure 1A**; p < 0.05). Using GLMs, we identified TAI as a risk factor for fertilization in euthyroid women undergoing IVF-ET (**Figure 1B**).

**Table 1:** A comparison of basic clinical characteristics between the TAI positive group and TAI negative group.

Characteristic	Age(years)	BMI (kg/m^2^)	Duration of infertility(years)	D3_FSH ( IU/L)	AMH (ng/mL)	Total AFC	FT4 (pmol/L)
TAI positive (n=565)	32.75 ± 5.02	22.47 ± 2.75	4.39 ± 3.24	4.65 ± 3.59	4.17 ± 3.28	13.59 ± 5.70	17.08 ± 4.74
TAI negative (n=3767)	32.07 ± 4.85	22.24 ± 2.77	4.21 ± 3.12	4.56 ± 7.28	4.60 ± 3.65	14.15 ± 5.62	16.51 ± 2.87
P-value	0.043	0.064	0.217	0.639	0.044	0.050	0.056

### 3.2 WGCNA identified TAI-associated miRNA modules in euthyroid women.

**Figure 2:**
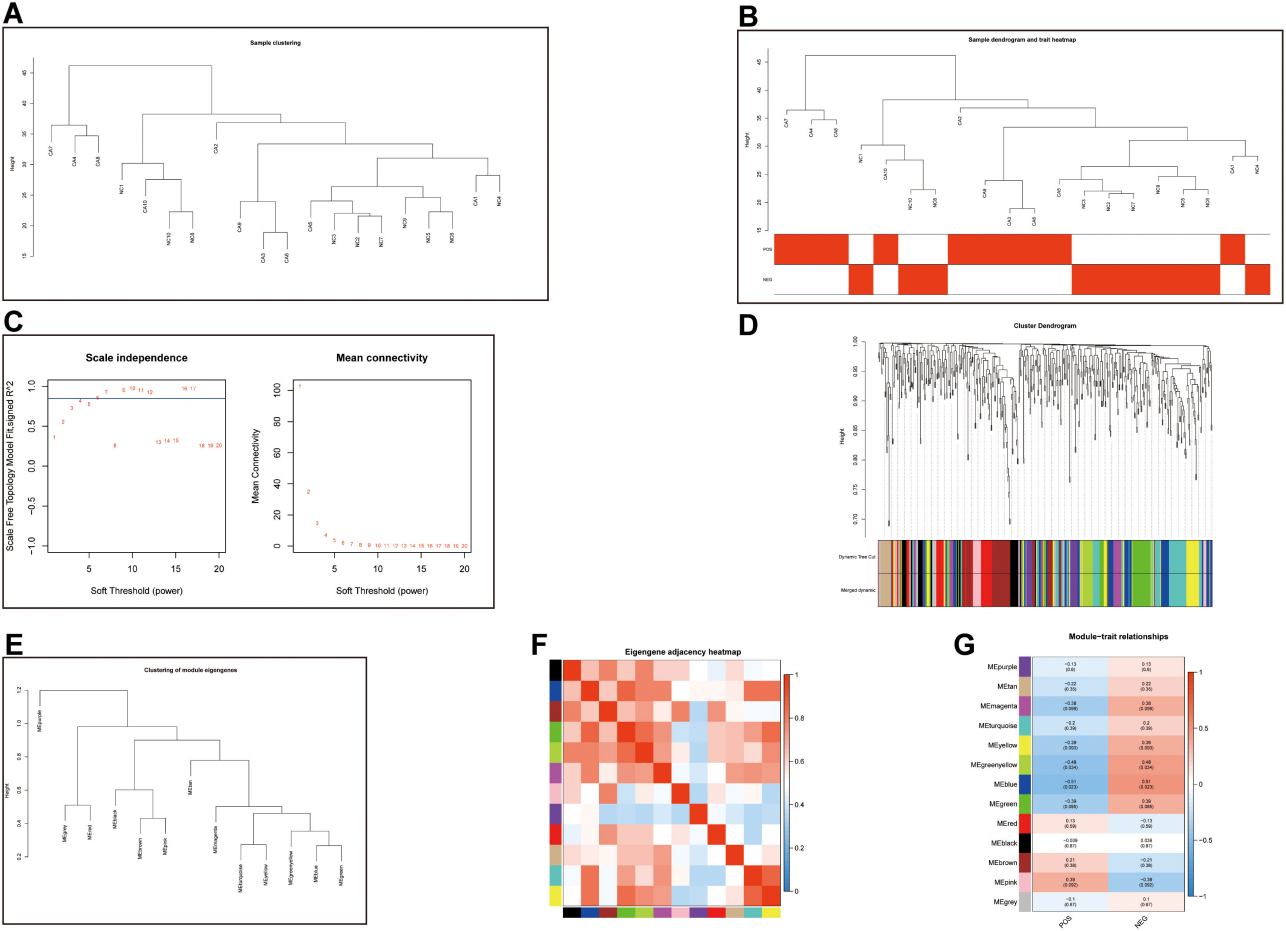
WGCNA of miRNA expression matrix associated with TAI in euthyroid women. **(A)** Sample clustering within the dataset, where branches represent samples and the vertical axis shows the height of hierarchical clustering. **(B)** Clustering results and phenotypic information, with the upper section showing clustering and the lower section displaying trait samples (red segments indicate trait samples). **(C)** Scale-free soft threshold distribution, with the horizontal axis representing weight parameter (power) values; the left vertical axis shows Scale-Free Topology Model Fit (signed R²), and the right vertical axis indicates the mean adjacency functions of all genes in each module. **(D)** Module clustering dendrogram displaying hierarchical clustering of genes into various modules, each represented by different colors. **(E)** Hierarchical clustering dendrogram of modules, where higher correlations are represented by closer clustering relationships among modules. **(F)** Heatmap of module–module correlations, illustrating the distribution of correlation patterns among different modules. **(G)** Heatmap of Module-Clinical Trait Associations, where the vertical axis represents modules, the horizontal axis represents traits, and each square indicates the correlation coefficient and significance P value for specific module-trait relationships.

Clustering analysis showed well-separated samples, with no exclusions (**Figure 2A**). A heat map integrating sample traits illustrated clustering and clinical characteristics (**Figure 2B**). The optimal power value in WGCNA was set at 6 (**Figure 2C**), ensuring a scale-free network. Co-expression analysis identified 13 gene modules (**Figure 2D**), with the blue and green-yellow modules (58 and 18 miRNAs, respectively) selected as key modules based on clinical correlation (**Figures 2E–G**).

### 3.3 Hub miRNAs were identified by intersecting miRNA modules and differentially expressed miRNAs in euthyroid women with TAI

**Figure 3:**
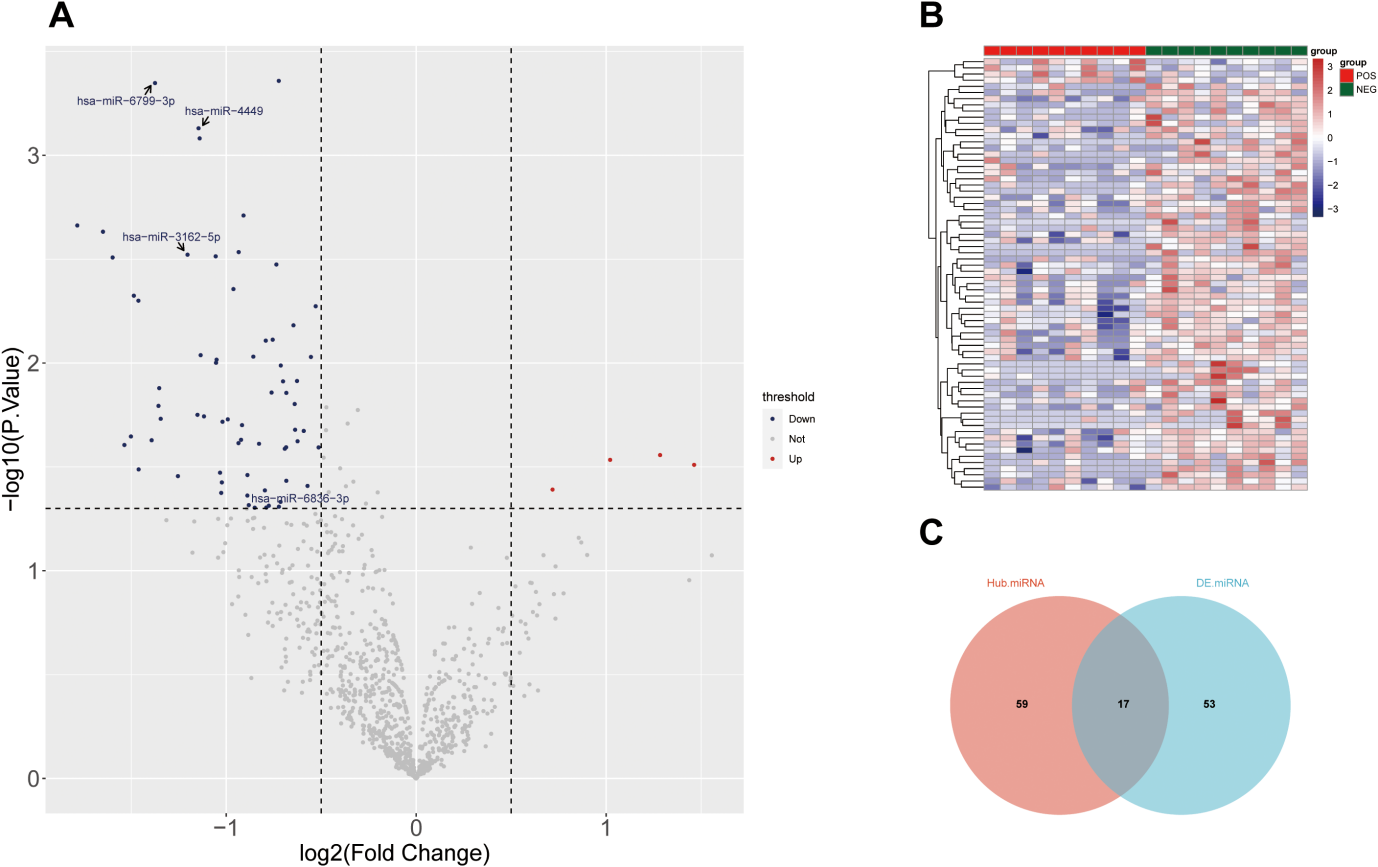
Expression of miRNAs of FF in euthyroid women with or without TAI undergoing IVF-ET. **(A)** Volcano plot comparing miRNAs in TAI(POS) and TAI-negative (NEG) patients. Red points indicate upregulated miRNAs, blue points indicate downregulated miRNAs, and gray points indicate no significant difference. **(B)** Heatmap of differentially expressed miRNAs between TAI-positive (POS) and TAI-negative (NEG). Each square represents a miRNA, with red indicating high expression and blue indicating low expression. The first row shows sample groupings, with green for TAI-negative samples and red for TAI-positive samples. **(C)** Venn diagram showing the intersection of differentially expressed miRNAs and hub miRNAs.

A total of 70 significant DE-miRNAs were identified between TAI positive (POS) and TAI negative (NEG) samples, including 4 upregulated and 66 downregulated miRNAs (**Supplementary Table 1**). Volcano and heat maps were generated to display the distribution of DE-miRNAs (**Figures 3A, B**). Seventeen intersecting miRNAs were identified by overlapping hub miRNAs with DE-miRNAs (**Figure 3C**).

### 3.4 Functional enrichment analysis of TAI-associated hub miRNAs in euthyroid women

**Figure 4:**
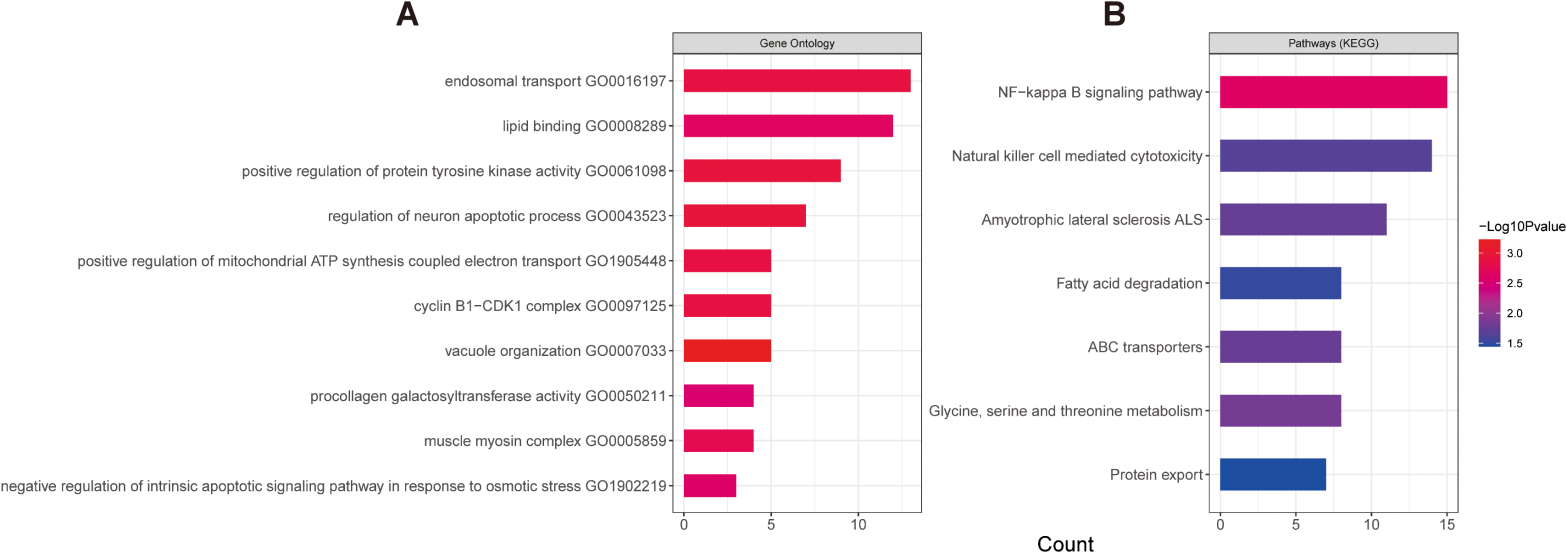
Functional enrichment analysis of DE-miRNAs in euthyroid women with or without TAI undergoing IVF-ET. **(A)** GO analysis of DE- miRNAs in euthyroid women with or without TAI undergoing IVF-ET. **(B)** KEGG analysis of DE-miRNAs in euthyroid women with or without TAI undergoing IVF-ET.

GO and KEGG enrichment analysis of the 17 intersecting miRNAs identified 226 GO terms and 7 KEGG pathways, including lipid binding, NF-kappa B signaling, cellular hyperosmotic response, glycine, serine, and threonine metabolism, fatty acid degradation, protein export, and regulation of neuron apoptosis. The top 10 GO and KEGG enrichment results are presented by P-value ranking (**Figure 4**).

### 3.5 Identification of TAI-associated critical miRNAs in euthyroid women using machine learning

**Figure 5:**
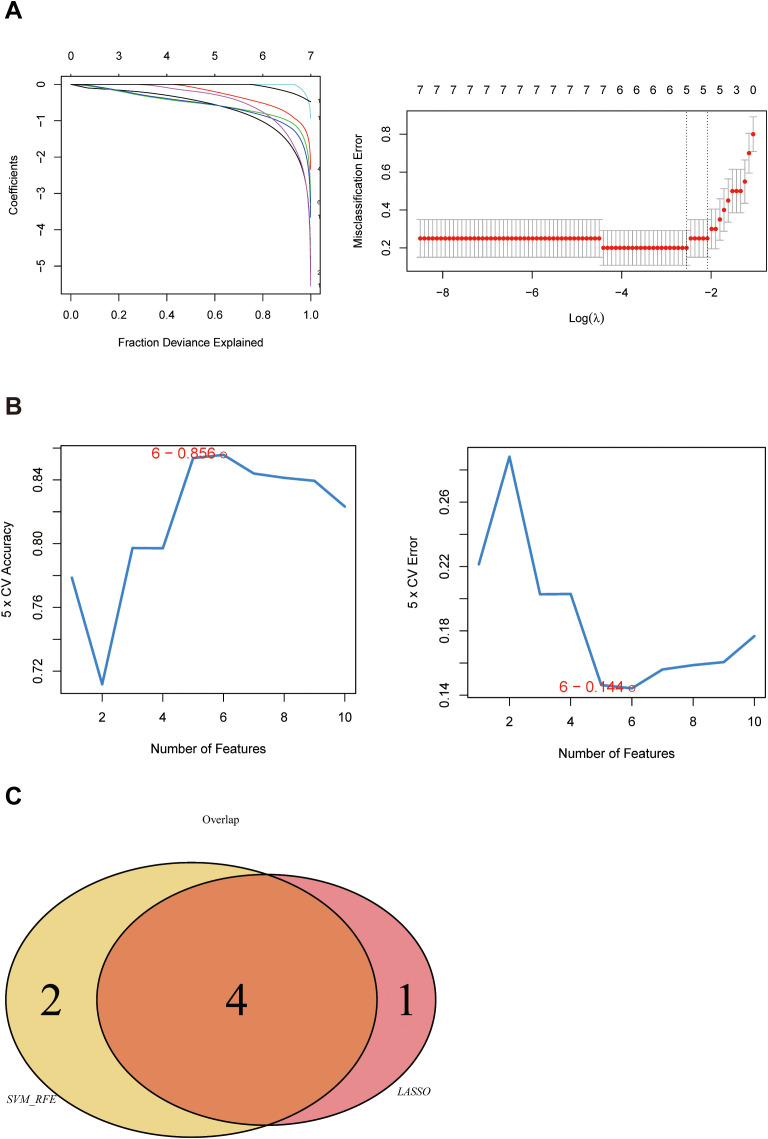
Identification of key miRNAs associated with TAI in euthyroid women undergoing IVF-ET using machine learning. **(A)** Characteristic miRNAs were screened using Lasso regression. **(B)** Characteristic miRNAs were identified using SVM. **(C)** Venn diagram illustrating the overlap of characteristic miRNAs identified through Lasso and SVM analyses.

LASSO regression analysis identified hsa-miR-6799-3p, hsa-miR-6836-3p, hsa-miR-4449, hsa-miR-6086, and hsa-miR-3162-5p as key miRNAs (**Figure 5A**). SVM analysis with 5-fold cross-validation selected hsa-miR-3162-5p, hsa-miR-6730-5p, hsa-miR-6799-3p, hsa-miR-4449, hsa-miR-4685-5p, and hsa-miR-6836-3p, achieving the highest model accuracy (**Supplementary Table 2**). **Figure 5B** shows the model accuracy for different feature sets. The intersection of miRNAs identified by both LASSO and SVM analyses revealed four critical miRNAs: hsa-miR-6799-3p, hsa-miR-6836-3p, hsa-miR-4449, and hsa-miR-3162-5p (**Figure 5C**).

### 3.6 Diagnostic value of critical miRNAs for euthyroid women with TAI

**Figure 6:**
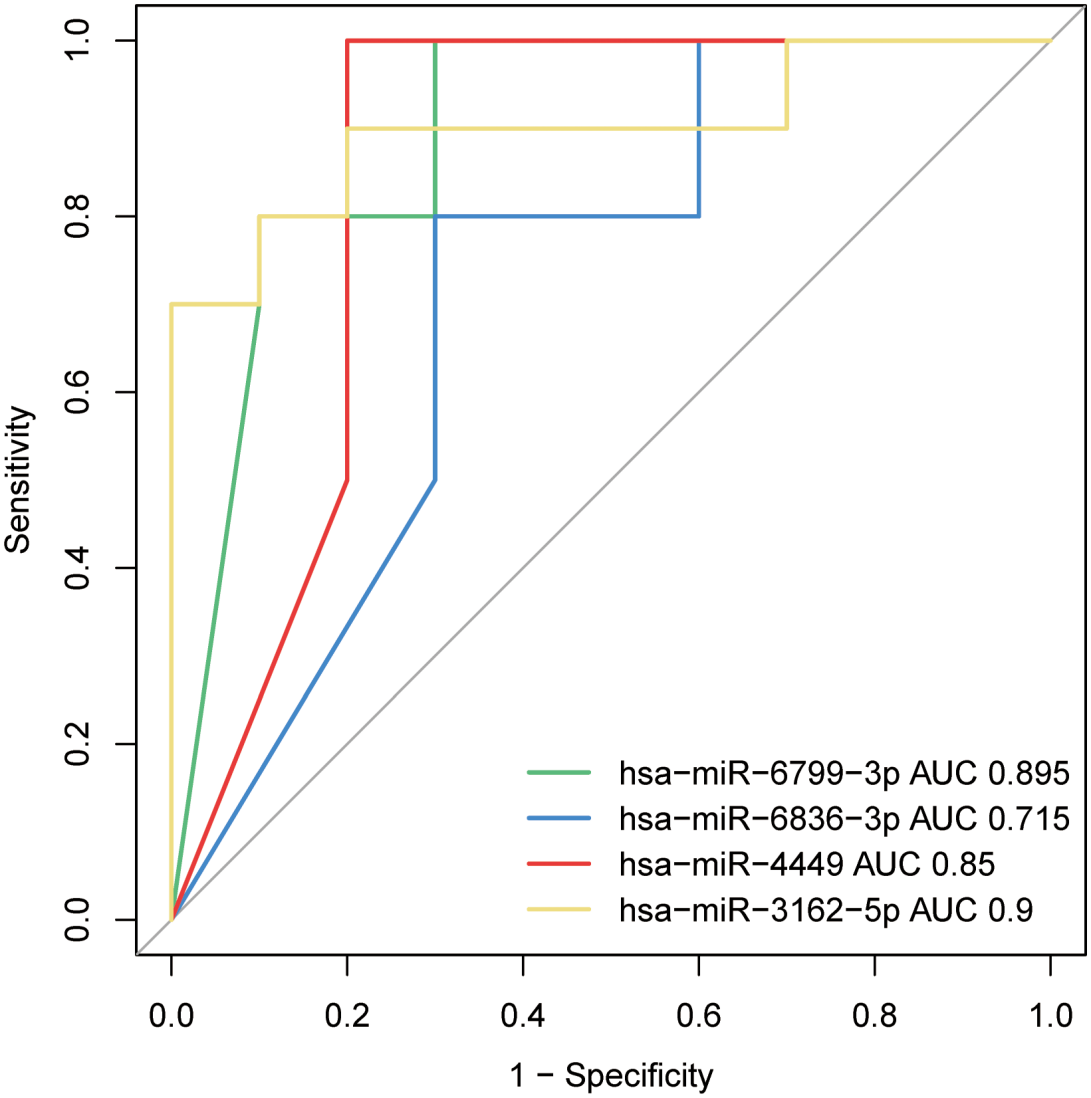
ROC curves of the key miRNAs were generated to evaluate their diagnostic potential for identifying euthyroid women with TAI undergoing IVF-ET.

ROC curve analysis showed that the AUC values for hsa-miR-6799-3p, hsa-miR-6836-3p, hsa-miR-4449, and hsa-miR-3162-5p exceeded 0.7 (**Figure 6**), demonstrating their effectiveness in distinguishing TAI-positive from TAI-negative samples.

### 3.7 Functional enrichment analysis of mRNAs associated with TAI in miRNA-mRNA pairs

**Figure 7:**
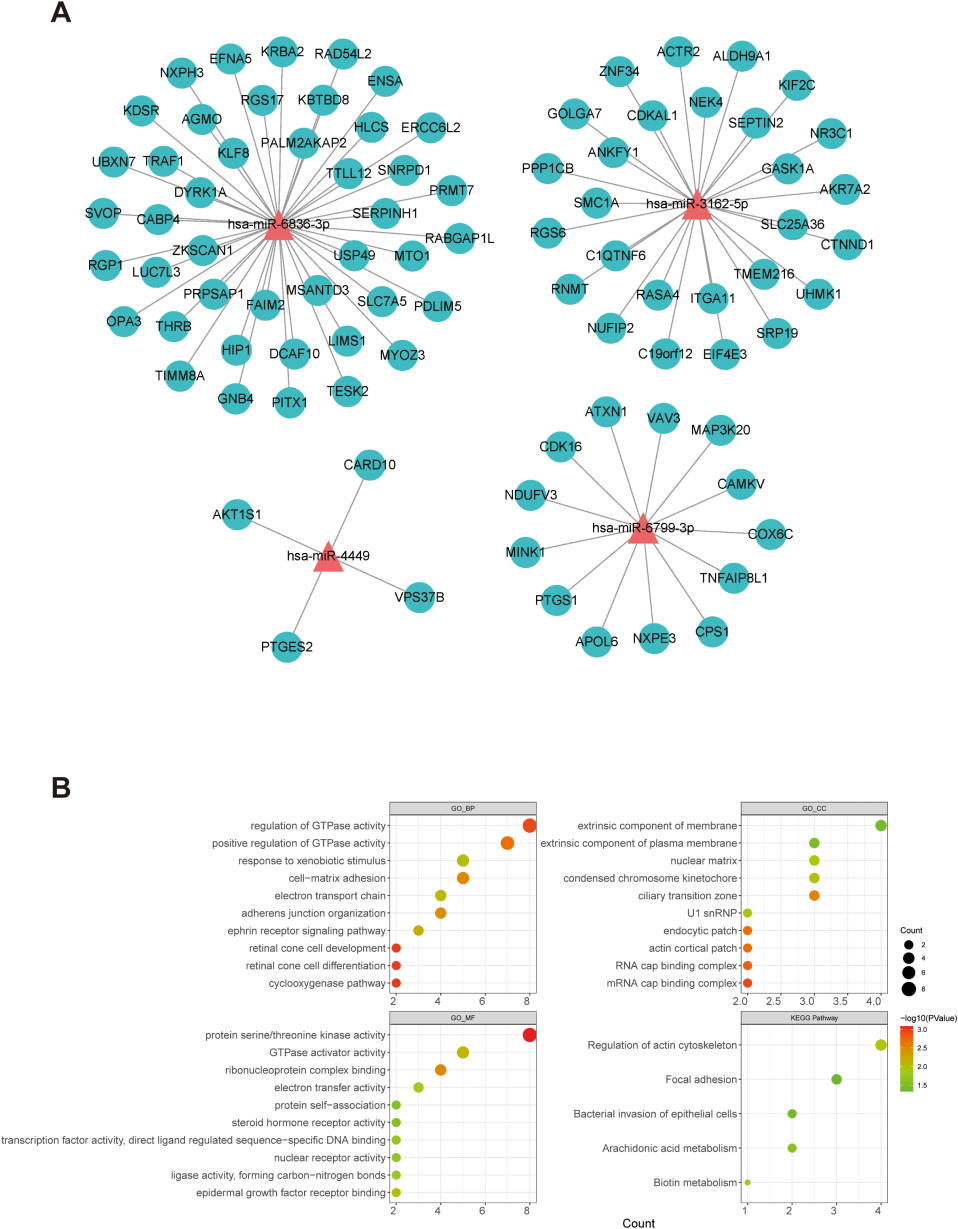
Functional enrichment analysis of mRNAs in miRNA-mRNA Pairs for euthyroid women with TAI undergoing IVF-ET. **(A)** Network diagram of miRNA-mRNA interactions, with red nodes representing key miRNAs and cyan nodes indicating predicted mRNAs. **(B)** GO and KEGG enrichment analyses were performed for mRNAs involved in the miRNA–mRNA interaction network.

The 4 miRNAs were paired with 87 target mRNAs, including hsa-miR-6836-3p-KDSR, hsa-miR-3162-5p-CDKAL1, hsa-miR-4449-PTGES2, and hsa-miR-6799-3p-VAV3. The miRNA-mRNA network was visualized to illustrate these relationships (**Figure 7A**). GO and KEGG enrichment analysis of the mRNAs in these pairs yielded 47 GO biological processes (BP), 22 cellular components (CC), 24 molecular functions (MF), and 5 KEGG pathways. Key pathways included cell-matrix adhesion, negative regulation of Target of Rapamycin (mTOR) signaling, DNA endoreduplication, synapse structure/activity regulation, and ephrin receptor signaling. The top 10 GO and KEGG enrichment results, ranked by P-value, are shown in (**Figure 7B**).

## 4 Discussion

Based on clinical data from 4,332 infertile women undergoing IVF-ET, this study found that, under euthyroid conditions, TAI-positive patients had a lower fertilization rate than TAI-negative patients, and multivariate analysis further indicated that TAI was a risk factor for impaired fertilization. By integrating follicular fluid exosomal miRNA sequencing, bioinformatics analysis, and machine learning approaches, four key miRNAs were identified: hsa-miR-6799-3p, hsa-miR-6836-3p, hsa-miR-4449, and hsa-miR-3162-5p. All four miRNAs showed AUC values greater than 0.7, suggesting their potential value in distinguishing TAI-positive from TAI-negative samples. Target gene enrichment analysis indicated that these miRNAs may participate in TAI-related reproductive impairment through pathways such as cell–matrix adhesion and mTOR signaling regulation. Overall, from both clinical outcome and molecular biomarker perspectives, our findings suggest that TAI is not a “biologically silent” condition in euthyroid infertile women, but may adversely affect early fertilization processes during IVF-ET.

Previous studies have reported inconsistent conclusions regarding whether TAI affects assisted reproductive outcomes. A systematic review and meta-analysis by Busnelli et al. found no clear adverse effects of TAI on the number of retrieved oocytes, fertilization, implantation, or clinical pregnancy outcomes in IVF/ICSI cycles (4). Similarly, a meta-analysis by Venables et al. focusing on euthyroid women suggested that the impact of TAI on pregnancy outcomes remains unclear (5). Bucci et al. also noted that, in the absence of overt thyroid dysfunction, the relationship between TAI and ART outcomes remains controversial (3). Nevertheless, some recent studies have suggested that TAI may affect oocyte yield, embryo quality, and even fertilization and embryonic development in specific subgroups (19–21). The present findings tend to support the hypothesis that TAI may influence early laboratory outcomes of ART. The discrepancies among previous studies may be attributable to differences in study populations, including whether participants were euthyroid or had diminished ovarian reserve, antibody positivity thresholds, IVF versus ICSI procedures, selected outcome measures, and the extent of adjustment for confounding factors.

The association between TAI and impaired fertilization observed in this study suggests that its adverse effects may occur at the follicular stage before embryo formation. In this regard, Monteleone et al. proposed the “ovarian follicle hypothesis,” suggesting that thyroid autoantibodies may enter the follicular fluid microenvironment and create a local immune milieu unfavorable for oocyte maturation and fertilization (6). Medenica et al. further demonstrated that thyroid autoantibody levels in follicular fluid were significantly correlated with serum levels in TAI-positive women, suggesting their potential influence on ART outcomes (8). In addition, proteomic and metabolomic studies have revealed altered protein expression and metabolic profiles in the follicular fluid of patients with TAI or Hashimoto’s thyroiditis, supporting the hypothesis that TAI may affect reproductive outcomes by disturbing the local follicular microenvironment (22, 23). Therefore, our finding that TAI is associated with a reduced fertilization rate is biologically plausible and suggests that autoimmune status should not be overlooked clinically merely because patients are euthyroid.

In this study, enrichment analysis revealed that the target genes of TAI-associated miRNAs were significantly enriched in pathways related to the mTOR signaling pathway and cell–extracellular matrix adhesion. The mTOR signaling pathway is a central hub integrating hormonal, nutritional, and energy signals and plays a multi-level regulatory role in follicular development and oocyte maturation. At the follicular level, the mTOR signaling pathway is widely involved in regulating multiple stages of folliculogenesis, from primordial follicle activation to preovulatory follicle development. Studies have shown that luteinizing hormone can activate the mTOR signaling pathway in preovulatory follicles, thereby balancing oocyte energy metabolism and regulating the ovulatory process (24). During early folliculogenesis, exosomes derived from human platelet lysate primarily promote primordial follicle activation through the PI3K/Akt signaling pathway (25). At the oocyte level, mTOR exerts stage-specific effects; appropriate activation of the mTOR signaling pathway is essential for meiotic resumption, cytoskeletal reorganization, and polar body extrusion, and its dysregulation may directly impair oocyte developmental competence and fertilization potential (26). In addition, a systematic review has highlighted that the mTOR signaling pathway plays a central role in female reproductive function, including ovarian activity, cyclic endometrial remodeling, embryo implantation, and placental formation (27). Therefore, the differentially expressed miRNAs identified in this study may interfere with normal intra-follicular signaling by regulating genes involved in the mTOR signaling pathway, representing a potential molecular mechanism through which TAI affects oocyte fertilization competence.

TAI may directly or indirectly affect the follicular microenvironment through immune-mediated mechanisms. The ovary and thyroid share multiple common antigens, including proteins such as peroxidases, which provides a molecular basis for the potential direct ovarian damage mediated by thyroid autoantibodies (22). Recent studies have shown that TPO is not only expressed in the thyroid gland but is also present on granulosa cells within follicular fluid, suggesting that TPOAb may act at the ovarian level by directly recognizing TPO antigens on granulosa cells, thereby impairing oocyte quality (21). At the level of the local immune microenvironment, abnormal distributions of immune cell subsets such as Th17 cells have been identified in the follicular fluid of TAI-positive patients. These Th17 cells may influence ovarian reserve function through the secretion of cytokines such as IL-17A (24). Taken together, the effects of TAI on the follicular microenvironment are likely multi-layered: thyroid autoantibodies may penetrate the blood–follicle barrier and directly act on ovarian tissues; systemic immune dysregulation associated with TAI may indirectly impair the functional state of granulosa and cumulus cells by altering the intra-follicular cytokine and chemokine milieu; and these immune abnormalities may further reshape the exosomal miRNA expression profile in follicular fluid, thereby disrupting key downstream biological pathways at the molecular level.

Follicular fluid–derived exosomes, as important mediators of intercellular communication, play a key role in maintaining follicular microenvironment homeostasis, and alterations in their miRNA cargo may represent an important bridge linking TAI-related immune abnormalities to impaired fertilization. Exosomal miRNAs encapsulated within extracellular vesicles are crucial mediators of paracrine communication among granulosa cells, cumulus cells, and oocytes (28). Follicular fluid extracellular vesicles mediate communication between the oocyte and somatic cells within the follicle and are essential for follicular development. Recent studies have further demonstrated that small extracellular vesicles in follicular fluid can significantly alter gene expression in granulosa cells related to extracellular matrix remodeling, cell cycle regulation, and Rho GTPase signaling pathways, all of which are critical for cumulus expansion and the normal function of the cumulus–oocyte complex (28). Functionally, exosomes secreted by granulosa and cumulus cells into follicular fluid carry miRNA cargos that dynamically change across follicular development stages, finely regulating gene expression in target cells and thereby supporting oocyte maturation, cumulus expansion, and acquisition of developmental competence (29). Based on the present findings, the systematic alteration of miRNA expression profiles in follicular fluid exosomes from TAI-positive patients may disrupt these key intercellular communication mechanisms, thereby impairing the normal regulation of critical biological processes such as the mTOR signaling pathway and cell–extracellular matrix adhesion within the follicle, ultimately affecting oocyte fertilization potential at the molecular level.

The clinical significance of this study lies in the finding that, even among euthyroid infertile women, TAI may indicate an increased risk of impaired fertilization and should therefore be considered during pre-IVF-ET evaluation. Meanwhile, key follicular fluid exosomal miRNAs may serve as novel molecular biomarkers for identifying TAI-related abnormalities in the follicular microenvironment. However, This study still has several limitations that should be considered when interpreting the findings. First, this was an exploratory study with a relatively small sample size (10 TAI-positive and 10 TAI-negative samples), which may limit statistical power and increase the risk of chance findings. Therefore, the differentially expressed miRNAs and potential biomarkers identified in this study should be regarded as preliminary candidates for screening and mechanistic exploration rather than direct clinical diagnostic tools, and their robustness needs to be further validated in larger independent cohorts.

Second, in the high-throughput miRNA sequencing analysis, we used a threshold of P < 0.05 and |log2FC| > 0.5 for screening without performing multiple-testing correction using false discovery rate (FDR). Although this approach helps reduce the risk of missing potentially biologically meaningful candidates under small-sample conditions, it may also increase the likelihood of false-positive results. Therefore, future studies should adopt more stringent statistical correction methods in larger cohorts to improve the reliability and reproducibility of the findings.

In addition, as this was a retrospective exploratory analysis, no qRT-PCR validation was performed in an independent cohort, and functional experiments were also lacking to further support the biological roles of the candidate miRNAs. Consequently, further validation in independent samples and functional studies using cellular and/or animal models is required to clarify their potential mechanisms in TAI-associated reproductive dysfunction.

Finally, regarding the machine learning analyses (LASSO and SVM), due to the limited sample size, these methods were primarily used for feature selection rather than for constructing robust predictive models. Therefore, there is a potential risk of overfitting, and systematic evaluation of model stability, repeated cross-validation, and external validation are still lacking. Future studies should further validate the robustness and reproducibility of the selected features in larger datasets.

## Data Availability

The exosomal miRNA sequencing data generated in this study have been deposited in the NCBI Sequence Read Archive (SRA) under accession numbers SRR32920729–SRR32920748. The clinical data of the participants cannot be publicly shared due to patient privacy protection (e.g., age, BMI, hormone levels, and IVF outcomes), but are available from the corresponding author upon reasonable request and approval from the Institutional Ethics Committee of the First People’s Hospital of Yunnan Province.

## Supplementary material

Supplementary Table 1 Identified 70 significant DE-miRNAs between TAI-positive and negative in euthyroid women.

Supplementary Table 2 features miRNAs were selected reaching the highest model accuracy.
