# Supplementary material for: Thyroid autoimmunity is associated with fertilization impairment and follicular fluid exosomal miRNA alterations in euthyroid women undergoing IVF-ET
Source: Front Immunol. 2026 Jul 10;17:1870650. doi: 10.3389/fimmu.2026.1870650 (PMC13395943; doi:10.3389/fimmu.2026.1870650)
Supplement: Supplementary Table 1 — Identified 70 significant DE-miRNAs between TAI-positive and negative in euthyroid women. [file DataSheet1.pdf]

Table S1 Identified 70 significant DE-miRNAs between POS and NEG samples

	logFC	AveExpr	t	P.Value	adj.P.Val	B
hsa-miR-4687-5p	1.46324246	11.363971	2.32524991	0.0308999	0.5327563	-3.4650134
hsa-miR-4668-5p	1.28403442	11.288572	2.37733042	0.0277259	0.504107	-3.3811254
hsa-miR-642a-3p	1.02111332	7.6968198	2.35186826	0.0292378	0.5129446	-3.4222516
hsa-miR-8085	0.71796554	7.8460596	2.19150838	0.0406486	0.5775703	-3.6760692
hsa-miR-12127	-0.5137811	7.0581865	-2.4178988	0.0254662	0.4787003	-3.3151647
hsa-miR-661	-0.5291264	7.1551093	-3.1327716	0.0053225	0.332654	-2.0857598
hsa-miR-378i	-0.5540971	7.1588143	-2.88161	0.0093392	0.4107425	-2.5294143
hsa-miR-6500-5p	-0.5720056	7.1605195	-2.2114393	0.0390363	0.5761959	-3.645035
hsa-miR-5688	-0.5919434	7.3081165	-2.5042643	0.0212155	0.4787003	-3.1730467
hsa-miR-6874-5p	-0.6246334	7.1779934	-2.4502893	0.0237865	0.4787003	-3.2621287
hsa-miR-4754	-0.6272524	7.2416763	-2.7606132	0.0121937	0.4534511	-2.7393106
hsa-miR-8073	-0.6373649	7.0865227	-2.5104318	0.0209389	0.4787003	-3.1628135
hsa-miR-4798-3p	-0.6397071	7.0876938	-2.6433541	0.0157412	0.4787003	-2.9397404
hsa-miR-939-5p	-0.6461007	7.2584982	-3.0387777	0.0065771	0.3868888	-2.2528752
hsa-miR-12120	-0.6836337	11.211978	-2.7001758	0.0139146	0.4638206	-2.843011
hsa-miR-4755-3p	-0.6837679	7.211168	-2.2393585	0.036876	0.568725	-3.6013096
hsa-miR-4691-5p	-0.6846621	7.3039035	-2.4184399	0.0254372	0.4787003	-3.3142814
hsa-miR-619-5p	-0.6917568	7.387816	-2.4107813	0.0258498	0.4787003	-3.3267752
hsa-miR-4634	-0.700966	12.334418	-2.7587641	0.0122432	0.4534511	-2.7424953
hsa-miR-4470	-0.7128314	7.3694102	-2.8387285	0.0102686	0.4107425	-2.6041298
hsa-miR-4745-5p	-0.713845	7.9062714	-2.1238117	0.0465889	0.5945098	-3.7803199
hsa-miR-3161	-0.7226776	7.5252122	-2.0969847	0.0491533	0.5945098	-3.8211216
hsa-miR-7706	-0.7233101	7.1294953	-4.2172541	0.000438	0.2071915	-0.1245902
hsa-miR-940	-0.7359683	7.4313861	-3.3363914	0.0033511	0.2792555	-1.7203848
hsa-miR-5585-5p	-0.7555114	7.3595663	-2.9676756	0.0077119	0.4102715	-2.3784712
hsa-miR-3922-5p	-0.7614101	7.317961	-2.7019432	0.0138612	0.4638206	-2.8399901
hsa-miR-9901	-0.7757725	10.719065	-2.1025809	0.048608	0.5945098	-3.8126348
hsa-miR-6836-3p	-0.7892862	7.5612222	-2.0920504	0.0496386	0.5945098	-3.8285939

hsa-miR-4738-3p	-0.7918594	7.224317	-2.9628637	0.0077952	0.4102715	-2.3869429
hsa-miR-2277-3p	-0.7965184	9.6975243	-2.1870634	0.0410164	0.5775703	-3.6829695
hsa-miR-663a	-0.8273448	11.724653	-2.4370891	0.0244581	0.4787003	-3.2837817
hsa-miR-6895-5p	-0.8499565	8.3461548	-2.0914492	0.049698	0.5945098	-3.8295035
hsa-miR-3973	-0.8573625	7.3537863	-2.8829849	0.0093108	0.4107425	-2.5270131
hsa-miR-942-3p	-0.8814734	8.2037473	-2.1058205	0.0482949	0.5945098	-3.8077159
hsa-miR-1233-5p	-0.8883551	7.8436832	-2.2705686	0.0345913	0.5556559	-3.5520892
hsa-miR-2861	-0.888732	7.8510898	-2.1586841	0.0434369	0.5869856	-3.7268445
ısa-miR-10522-5ţ	-0.9091124	7.2223965	-3.5725126	0.0019491	0.2792555	-1.2929659
hsa-miR-4690-5p	-0.9167649	7.2867697	-2.5343239	0.019899	0.4787003	-3.1230692
hsa-miR-3175	-0.9218608	7.9725073	-2.4580718	0.0233986	0.4787003	-3.2493377
hsa-miR-7158-5p	-0.9345349	7.2869646	-3.3966101	0.0029199	0.2792555	-1.6116618
hsa-miR-4685-5p	-0.9356051	7.9561186	-2.440395	0.0242883	0.4787003	-3.2783639
hsa-miR-6775-3p	-0.9620393	7.4298804	-3.2167953	0.0044002	0.332654	-1.9354753
hsa-miR-4517	-0.9917962	8.1218505	-2.5644595	0.0186566	0.4787003	-3.0727126
hsa-miR-6846-5p	-1.0196512	8.2304263	-2.5519776	0.019162	0.4787003	-3.0936002
hsa-miR-4725-3p	-1.0231802	7.9428981	-2.2306753	0.0375359	0.568725	-3.61494
hsa-miR-4741	-1.0263416	10.230822	-2.1734396	0.0421626	0.5775703	-3.7040716
hsa-miR-7107-5p	-1.032582	9.4249962	-2.2830656	0.0337135	0.5526802	-3.5322818
hsa-miR-4722-3p	-1.0509093	7.9437459	-2.8678733	0.0096278	0.4107425	-2.553386
hsa-miR-6753-5p	-1.0536476	7.862481	-2.8523805	0.0099635	0.4107425	-2.5803804
hsa-miR-8072	-1.0555334	8.0258493	-3.3761636	0.0030598	0.2792555	-1.6486043
hsa-miR-6738-5p	-1.1172302	8.6400385	-2.579783	0.0180532	0.4787003	-3.0470127
hsa-miR-6730-5p	-1.1345997	8.5071545	-2.8905164	0.0091566	0.4107425	-2.5138536
hsa-miR-6086	-1.1399299	7.8800028	-3.9422273	0.0008288	0.2071915	-0.6215276
hsa-miR-4449	-1.1456128	7.6173443	-3.9905468	0.0007409	0.2071915	-0.5339513
hsa-miR-5088-5p	-1.1514317	8.7566654	-2.587919	0.0177403	0.4787003	-3.0333419
hsa-miR-3162-5p	-1.2037668	9.1129172	-3.3839701	0.0030056	0.2792555	-1.6345028
hsa-miR-638	-1.2542997	11.043039	-2.2647608	0.0350063	0.5556559	-3.5612754
hsa-miR-6085	-1.3450121	8.5138889	-2.5673087	0.0185429	0.4787003	-3.0679389

hsa-miR-4758-3p	-1.3528011	8.5082503	-2.7243513	0.0132002	0.4638206	-2.8016275
hsa-miR-4749-3p	-1.3567257	9.4345617	-2.6338521	0.016068	0.4787003	-2.9558396
hsa-miR-6799-3p	-1.3748738	7.734513	-4.2067743	0.0004487	0.2071915	-0.1434445
hsa-miR-6743-3p	-1.3927048	9.60885	-2.4555496	0.0235236	0.4787003	-3.2534851
hsa-miR-1470	-1.4612563	10.353253	-2.3008301	0.032501	0.5416834	-3.5040298
hsa-miR-6089	-1.4622557	9.6588533	-3.1599673	0.0050051	0.332654	-2.0372028
hsa-miR-4484	-1.4863982	9.004783	-3.1844947	0.0047347	0.332654	-1.9933385
hsa-miR-5589-3p	-1.5016275	9.3974261	-2.475381	0.022557	0.4787003	-3.2208238
hsa-miR-5787	-1.5361644	10.18288	-2.4307696	0.0247859	0.4787003	-3.2941289
hsa-miR-6768-5p	-1.5982029	9.5034617	-3.3704898	0.0030998	0.2792555	-1.658851
hsa-miR-4726-5p	-1.6487986	9.8475285	-3.4945475	0.0023322	0.2792555	-1.4343851
hsa-miR-9899	-1.7840829	11.005868	-3.5244848	0.002177	0.2792555	-1.3801076

Note:POS: positive; NEG: negative
