# Supplementary material for: Thyroid autoimmunity is associated with fertilization impairment and follicular fluid exosomal miRNA alterations in euthyroid women undergoing IVF-ET
Source: Front Immunol. 2026 Jul 10;17:1870650. doi: 10.3389/fimmu.2026.1870650 (PMC13395943; doi:10.3389/fimmu.2026.1870650)
Supplement: Supplementary Table 2 — features miRNAs were selected reaching the highest model accuracy. [file DataSheet2.pdf]

Table S2 Six feature miRNAs were selected reaching the highest model accuracy

	FeatureName	FeatureID	AvgRank
1	hsa-miR-3162-5p	16	1.8
2	hsa-miR-6730-5p	10	4.2
3	hsa-miR-6799-3p	2	5
4	hsa-miR-4449	6	6.6
5	hsa-miR-4685-5p	17	6.8
6	hsa-miR-6836-3p	4	7.8
7	hsa-miR-6086	11	8.6
8	hsa-miR-4758-3p	13	8.6
9	hsa-miR-6089	3	9.6
10	hsa-miR-4484	8	9.8
11	hsa-miR-4517	12	11.2
12	hsa-miR-5589-3p	5	11.8
13	hsa-miR-7107-5p	9	11.8
14	hsa-miR-1233-5p	15	11.8
15	hsa-miR-6846-5p	1	12.4
16	hsa-miR-663a	14	12.4
17	hsa-miR-3175	7	12.8
